# Characterization of Gallium Indium Phosphide and Progress of Aluminum Gallium Indium Phosphide System Quantum-Well Laser Diode

**DOI:** 10.3390/ma10080875

**Published:** 2017-07-28

**Authors:** Hiroki Hamada

**Affiliations:** Department of Electric and Electronic Engineering, Kinki University, Higashi-Osaka 577-8502, Japan; h.hamada@ele.kindai.ac.jp

**Keywords:** GaInP, AlGaInP, epitaxial layer, metal organic chemical vapor deposition, misorientaion substrate, ordering structure, disordering structure, photoluminescence, strain compensated quantum well, semiconductor laser diode, light emitting diode, quantum dots, InAlAs, InP, InAs, GaAs(N), GaAs, InGaAs, GaAsP, thermal resistance

## Abstract

Highly ordered gallium indium phosphide layers with the low bandgap have been successfully grown on the (100) GaAs substrates, the misorientation toward [01−1] direction, using the low-pressure metal organic chemical vapor deposition method. It is found that the optical properties of the layers are same as those of the disordered ones, essentially different from the ordered ones having two orientations towards [1−11] and [11−1] directions grown on (100) gallium arsenide substrates, which were previously reported. The bandgap at 300 K is 1.791 eV. The value is the smallest ever reported, to our knowledge. The high performance transverse stabilized AlGaInP laser diodes with strain compensated quantum well structure, which is developed in 1992, have been successfully obtained by controlling the misorientation angle and directions of GaAs substrates. The structure is applied to quantum dots laser diodes. This paper also describes the development history of the quantum well and the quantum dots laser diodes, and their future prospects.

## 1. Introduction

Aluminum gallium indium phosphide (AlGaInP) laser diodes, which are operated at continuous-wave (CW) under room temperature conditions, were been developed by three Japanese companies in 1986 [1,2,3]. It was confirmed that the oscillating wavelength for each laser is longer than that calculated from theoretical bandgap, even though gallium indium phosphide (Ga_x_In_1−x_P) epitaxial layer with same composition are used. By this phenomenon, the development of the lasers and light emitting diodes (LEDs) operating at <650 nm have been prevented. The issues have been studied by many researchers since 1986. It was found that the phenomena are generated by (01−1) micro-steps on the surface of GaAs (100) substrates during the crystal growth processes using the metal organic chemical vapor deposition (MOCVD) method [4,5,6]. Then, many companies and researchers proposed the ordering suppressing technologies such as low III/V ratio [7], high temperature growth [8,9], high growth rate [10], Zinc (Zn) doping and diffusion [11,12], introduction of (111)A and (111)B plane; (110) crystal plane [13,14] and misorientation substrates [15,16,17,18,19,20,21,22]. Finally, it was introduced (100) GaAs substrates having misorientation angle towards [0 1 1] direction, which was discovered by Hamada’s team in 1988 [15], and technologies using these substrates have been widely used in production for over 25 years. Watt-class high power laser diodes, having strained quantum wells oscillating at 630 nm band, have been developed using the broad area structure and the misorientation substrates [23]. In 1995, 12 W high power laser diodes oscillating at 640 nm were also obtained by combining the array structure and silicon microchannel cooler [24]. Then, highly reliable watt-class laser diodes for projector applications were successfully developed by applying the laser structure with a lower thermal resistance in 2005 and 2006 [25,26]. Vertical cavity surface emitting lasers (VCSELs) oscillating at continuous wave (CW) have been successfully developed using the misorientation substrates [27,28,29]. Quantum dots (QDs) laser diodes have been aggressively studied using InAs/InGaAlAs and InAsP/AlGaInP materials. The laser diodes, which are capable of tuning the oscillating wavelength by changing the QD size, have been obtained [30,31]. The red laser diodes have been mainly applied as the light sources of measurement tools, DVDs, projectors and displays [32]. Recently, lasers are introduced in the field of medicine and agriculture. The applications were reported by Hamada in 2015 [32]. On the other hand, high efficiency orange and red color light Emitting diodes (LEDs) have been manufactured for many applications such as traffic signals, cars, and digital signage.

This paper describes some phenomena that have not yet been reported on the characterizations of GaInP grown on (100) GaAs substrates having misorientation angles by the MOCVD method, and also shows the characteristics of high performance strain compensated multiple quantum well (SC-MQW) lasers, which are grown on the substrates. This paper also discusses the development history of SC-QW structure and future development target.

## 2. Experiment

Gallium indium phophide (GaInP) epitaxial layers were grown on (100) gallium arsenide (GaAs) substrates having misorientation angle towards [011], [01−1] direction and (100) just using the low pressure MOCVD system with a load rock chamber. The heating of substrates was carried out by the RF induction method. Source materials are trimethylindium (TMI), trimetylgallium (TMG), methylaluminium (TMA), PH_3_, and AsH_3_. Dimethylzinc (DMZ), and SiH_4_, and H_2_Se as the doping sources were used for p- and n-type layers, respectively. The growth temperature and total pressure were 650–680 °C and 9.3 × 10^3^ Pa, respectively. V/III ratio was ~550, and the growth rate was 1.2 µm/h. The lattice mismatch of (Al*_x_*Ga_1−*x*_)InP epitaxial layer to GaAs substrates (Δa_⊥_/a_⊥_) was less than 2 × 10^−3^. The photoluminescence spectra were measured using the 488 nm Ar + laser, which is focused to spot size of approximately 250 µm^2^. The excitation light density is 8 W/cm^2^. The luminescence is dispersed by a monochrometer (SPEX 1000 M, HORIBA JOBIN YVON S.A.S., Edison, NJ, USA), detected using a cooled photomultiplier, and a lock-in amplifier. The wavelength scanning step and slit width of the monochrometer were 0.1–0.5 nm and 0.1–0.5 mm, respectively. Sample temperature was controlled using a helium cryostat system with heater in the ranges of 10–300 K.

## 3. Results and Discussion

### 3.1. GaInP Epitaxial Layer

The ordering phenomena of GaInP epitaxial layers grown on GaAs substrates were reported by Suzuki et al. and Gomyo et al. [4,5,6]. Then, many researchers aggressively studied suppressing the ordering [7,8,9,10,11,12,13,14,15,16,17,18,19,20,21,22]. As a result, during 1989–1994, it was recognized that the (100) GaAs substrates having misorientation towards [011] direction were useful to suppress the ordering generation and to develop high performance quantum well (QW) AlGaInP laser diodes. On the other hand, it was believed that the crystallinity of ordered GaInP epitaxial layers were not good in comparison to that of disordered ones, as the ordered GaInP epitaxial layers are constructed by two domains, such as [11−1] and [1−11] directions, and their micro-grain boundaries. The considerations have also been supported by many experimental results [4,5,6,17]. However, there is a possibility that highly ordered GaInP epitaxial layers having either [11−1] or [1−11] direction may have same characteristics as disordered ones. This possibility was pointed out by Schneider et al. in 1992 [33]. However, the proofs based on the experimental results have not been reported during 25 years. In this paper, highly ordered GaInP layers toward [11−1] direction have been successfully formed using GaAs (100) substrates having 5° misorientation toward [01−1] direction using the MOCVD method. The characterizations are described in the following section.

### 3.2. GaInP Epitaxial Layers

The characterizations of all samples, which are prepared for evaluations of GaInP epitaxial layers are summarized in Table 1. The samples are grown on four kinds of substrates. Each sample is characterized by sample number. Ex144, which is grown on (100) substrate, has the ordered structures of two directions toward [11−1] and [1−11] directions. Ex146 and Ex145-A are grown on 5° misorientation substrates at growth temperature of 650 °C and 680 °C, respectively. Ex145-B, which are grown on GaAs substrates with 5° misorientation [01−1] direction, has the ordered structure with an orientation of [11−1] direction. Ex145-A and Ex148 are characterized by almost and completely disordered structures, respectively.

**Figure 1:**
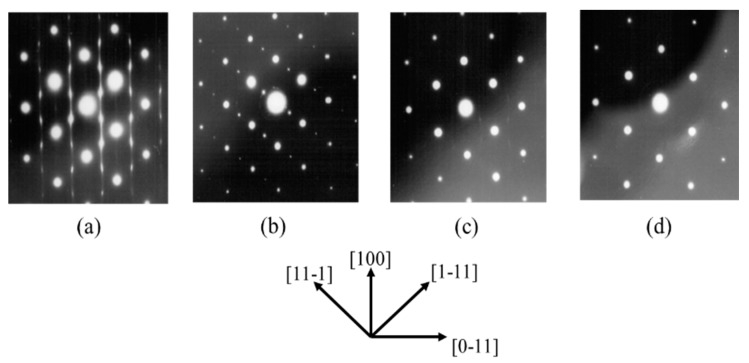
Transmission electron diffraction (TED) patterns of GaInP epitaxial layers for: (**a**) Ex144; (**b**) Ex145-B; (**c**) Ex145-A; and (**d**) Ex148.

Figure 1 shows transmission electron diffraction (TED) patterns of GaInP epitaxial layers grown on the GaAs (100) substrates, GaAs (100) substrates having 5° misorientation toward [01−1] and [011] direction and having 9° misorientation toward [011] direction. The sub spots of ($\frac{1}{2}\frac{1}{2}-\frac{1}{2}$) and ($\frac{1}{2}-\frac{1}{2}\frac{1}{2}$), which reflect the ordered structure having two directions, are observed for GaInP layers grown on GaAs (100) substrates, as shown in Figure 1a. Figure 1b shows the highly ordered structure without the sub spots ($\frac{1}{2}\frac{1}{2}-\frac{1}{2}$) toward [11−1]. On the other hand, the mixed structure having ordered and disordered arrangements is shown in Figure 1c. Figure 1d shows GaInP layers grown on 9° misorientation substrates toward [011] direction. This shows the completely disordered structure. The results are consistent with many reports [34,35]. However, the ordering dependence of the misorientation angle is stronger than those of previous reports [4,5,6,18]. These samples are used to characterizing the ordered and disordered structures.

**Figure 2:**
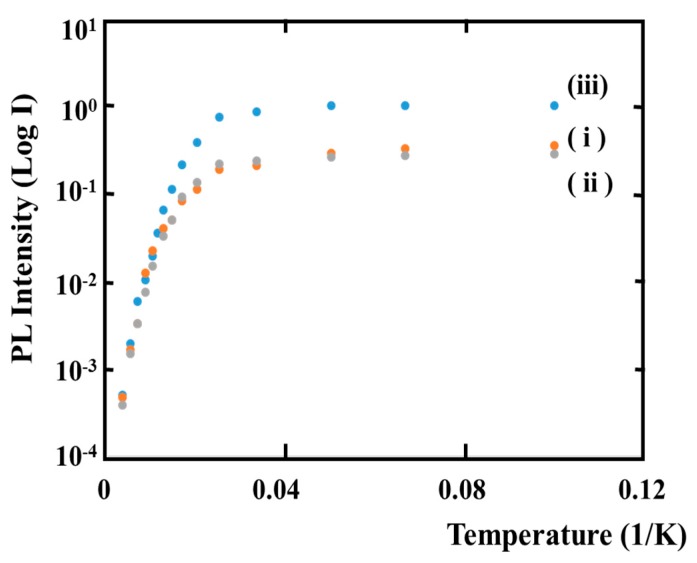
Temperature dependence of photoluminescence (PL) peak intensity for: (**i**) Ex145-B; (**ii**) Ex145-A; and (**iii**) Ex146.

Figure 2 shows the temperature dependence of photoluminescence (PL) peak intensity of ordered and disordered InGaP layers. Ex146, the Ex145-A and Ex145-B samples are used for the characterizations by the PL method. Temperature dependence of the PL emission efficiency (*η*) is given by the following equation [36].

*η*(*T*) = *P_r_*{*P_r_ + ^A^P_nr_ + ^B^P_nr_*}^−1^ (1)

where *P_r_* is the probability for radiative transition, which is independent for temperature. *^A^P_nro_* and *^B^P_nr_* are the probabilities for two non-radiative recombination mechanisms, as shown in the following equations:

*^A^P_nr_ = ^A^P_nro_ exp*(−*E_A_/kT*) (2)

*^B^P_nr_ = ^B^P_nro_ exp*(−*E_B_/kT*) (3)

where *E_A_* and *E_B_* are the thermal activation energies, and *^A^P_nro_* and *^B^P_nro_* are the temperature independent factors. *η*(*T*) is rewritten using Equations (2) and (3) as following equation.

*η*(*T*) = {1 + *^A^C exp*(−*E_A_/kT*) + *^B^C exp*(−*E_B_/kT*)}^−1^ (4)

where *^A^C = ^A^P_nro_/P_r_* and *^B^C = ^B^P_nro_/P_r_* are the ratio of non-radiative to radiative recombination probabilities. In the paper, *^A^C* and *^B^C* are estimated by fitting at the regions of low (10 K–100 K) and high (100 K–300 K) temperatures, respectively. The symbols of *k* and *T* are Boltzmann constant and temperature, respectively. Parameters such as *^A^C*, *E_A_*, *^B^C* and *E_B_* in Equation (4) are obtained by fitting to the experimental results in Figure 2. Table 2 shows the fitting parameters for each sample. The lowest column also shows the values for the ordered GaInP layer grown on (100) GaAs substrates, which was reported by Lambkin et al. *E_A_* includes, in general, localized energy and binding energy of the exciton. *E_A_* for all samples also lies in the spatial variation (4.5–30 meV) of band-edge minima, which was suggested for the disordered GaInP layers by Delong et al. [37]. The results for *^B^C*, which, for all samples, are virtually the same, mean that the non-radiative mechanisms in the region of high temperature (100 K–300 K) are basically not different between the highly ordered and disordered structures. From these results, the crystallinity of Ex145-B is the same as those of Ex145-A and Ex146. Especially, the *^A^C* of highly ordered LC159, which was reported by Lambkin et al. [36], is larger than that of Ex145-B. This means that LC159 has many non-radiative centers in comparison to that of Ex145-B. This may be attributed to the crystalline structure differences that LC159 and Ex145-B have, i.e., the ordered structure of two directions and one direction, respectively. In other words, this means that the number of domain boundaries in LC159 is much more than those of Ex145-B. It is also understood by the experimental results that the full width at half maximum (FWHM) of PL spectrum of Ex145-B at 10 K is one-half narrower than that of that of LC159. The results are also supported by the experimental results, which show that some deep levels generated by incorporating of oxygen are reduced using the misorientation substrates [38].

**Figure 3:**
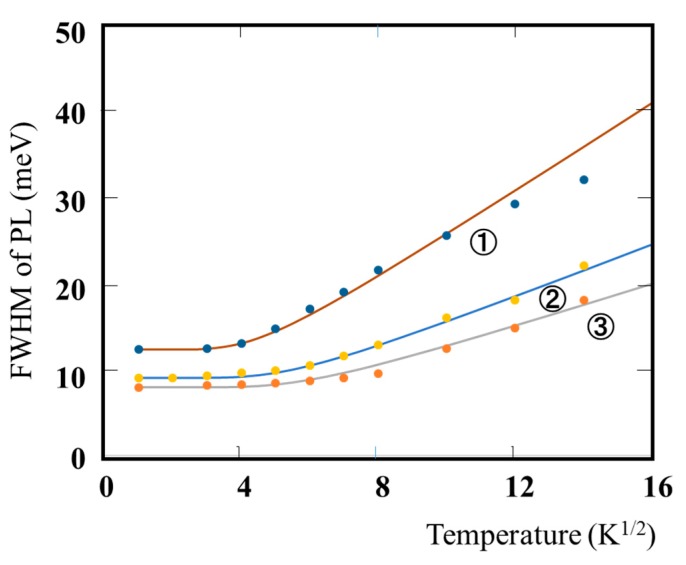
Temperature dependence of full width at half maximum (FWHM) of PL spectrum for: ① Ex145-B; ② Ex145-A; and ③ Ex146. Solid line is a fit to Equation (5) for each data.

Figure 3 shows the dependence of full width at half maximum (FWHM) of PL spectrum on temperature (1/T). For this experiment, Ex-146, and Ex145-A and -B samples are used. The FWHM (*W*), in general, is given by the configurational-coordinate model equation [39]:

*W = A* (*cothℏω*/2*kT*)^1/2^ (5)

where *A* is a constant whose value is equal to *W* as the temperature approaches 0 K, and *ℏω* is the energy of the vibration mode of the excited state. In this paper, Equation (5) has been fitted to the experimental values for each sample. The results are listed in Table 3. Equation (5) is consistent with each sample at less than 100 K. *A* and *ℏω* for each sample are virtually constant. *A* of Ex146-B, having the ordered structure, is larger than that of Ex145-A and Ex146. However, the value lies in the ranges of FWHM of PL for the disordered structure, which were reported by some papers [14,33]. From the results, it can be concluded that the differences between *A* and *ℏω* of the samples having the ordered structure (such as the single domain structure) toward either [11−1] or [1−11] direction and those of disordered ones is small.

**Figure 4:**
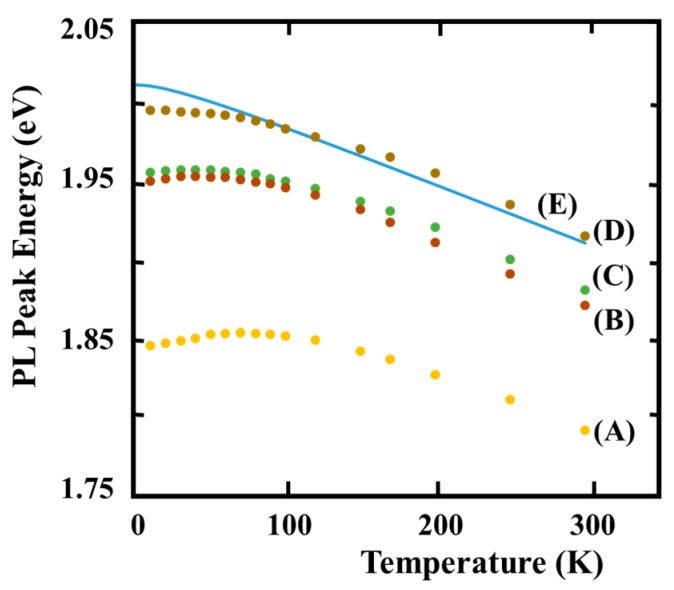
Temperature dependence of PL peak energy for GaInP epitaxial layers: (**A**) Ex145-B; (**B**) Ex145-A; (**C**) Ex146; and (**D**) Ex148. Solid line (**E**) is a calculation result based on Varshni equation [40] and Delong’s bandgap value [37].

**Figure 5:**
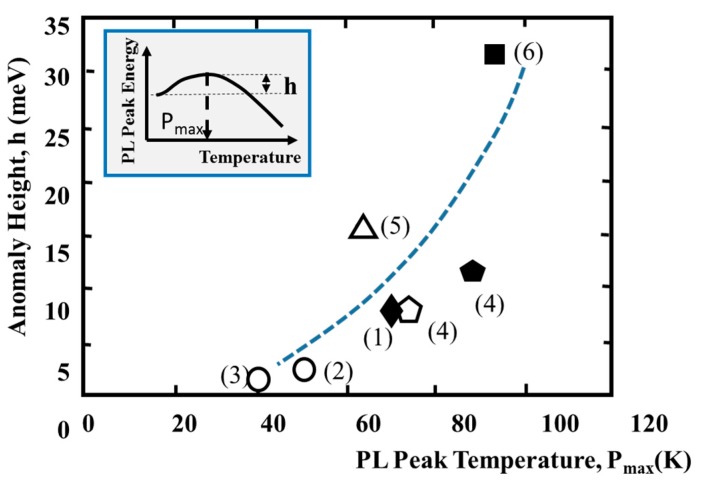
Relationship between anomalous height and PL peak temperature: (**1**) Ex145-B; (**2**) Ex145-A; (**3**) Ex146; (**4**) Ishitani et al. [42]; (**5**) Yanagisawa et al. [46]; and (**6**) Kondow et al. [43]. Solid symbols; Ordered structure, Circular symbols; Disordered + Ordered structure.

Figure 4 shows the dependence of bandgap energy on temperature (1/*T*). The samples are Ex148 having completely disordered, Ex146 having almost disordered, Ex145-A having mixed (the disordered + ordered) and Ex146-B having highly ordered structures. The solid line shows a fit to Varshni’s equation for the disordering structure [40,41]. The accuracy of the simulation increases by adopting the effect of thermal expansion, electron-phonon coupling, and electron-acoustic-phonon coupling [42]. The dependence of the disordered GaInP layers grown on 9° misorientation substrates basically meets Varshni’s equation. On the other hand, the dependence of PL spectrum for the ordered GaInP layers shows the anomalous characteristics at <100 K. The characteristics were also reported on the GaInP layers having the ordered structure grown on GaAs (100) substrates by Kondow et al. [43,44,45,46]. The ordered structure have two directions: [11−1] and [1−11]. On the other hand, the samples are GaInP layers having the ordering structure toward [11−1] direction. The PL peak energy shows 1.791 eV at room temperature, as shown in Figure 5, and is the lowest ever reported, to our knowledge [41]. The value may be attributed to the phenomenon that the ordered structure having atomic arrangement of one direction is enhanced by increasing (111)B micro-steps that appear on the surface by introducing the misorientation substrates toward [01−1] direction. On the other hand, PL energy of the completely disordered structure shows 1.914 eV. The PL peak energy difference between the highly ordered and completely disordered structure, which was reported by Delong et al., is as high as 166 meV at the low temperature condition. On the other hand, the differences are 130 meV at room temperature. The values are larger than those of the previous reports [4,5,6,33].

Figure 5 shows the relationship between the changing width “h” at anomaly region (see Figure 5) and temperature, which presents anomalous characteristics. The solid and circular symbols show the ordered and the disordered structures, respectively. The “h” values are also plotted using data in previously published papers [43,44,45,46]. The “h” values decrease with temperature, in which the anomalous characteristics are observed. The phenomena enable understanding that the anomalous characteristics are not observed for completely disordered GaInP layers. From the results, it is confirmed that the disordered area, which includes Ex146, is larger than those of Ex145-A. The ordered structures reported previous papers have two kinds of domains, towards [1−11] and [11−1] directions, showing the dependence of the anomalous height “h” on the temperature “P_max_”. The phenomenon may be supported by the considerations that the domain boundaries act as regions for relieving the stress in the layers.

Table 4 summarizes the characterizations of the highly ordered and ordered GaInP epitaxial layers reported in previous papers [42,43]. The PL peak-energies (band gap) of the ordered structures reported are 1.83–1.86 eV at 300 K. The band gap of Ex145-B is 1.791 eV. The values are the smallest ever reported, to our knowledge. The largest “h” value is obtained for GaInP layers grown on GaAs (100) substrates. The “h” of Ex145-B is smaller than those on the GaAs (100) substrates reported. FWHM of PL spectrum at low temperature of samples grown on the misorientation substrates is about one-half narrower than that grown on (100) substrates. The phenomenon that a PL spectrum with two peaks appears for the ordered samples grown on (100) substrates at below 30 K has been reported by Kondow et al. [43]. However, phenomena like this one are not observed for misorientation substrates. It is concluded that Ex145-B is better than those grown on the GaAs (100) substrates and have structures such as the single domain. Furthermore, this conclusion is supported by a report, in which the generation of deep levels in GaInP layers is suppressed using misorientation substrates [38]. High performance unicompositional devices that combine highly ordered and the disordered structures will be developed in the future [47].

### 3.3. Quantum Well AlGaInP Laser Diode

This section reviews quantum well AlGaInP laser diodes. The ordered structure controlling technologies, which were described in Section 3.2, are also useful to obtain the epitaxial layer with a smooth surface at atomic order. It is considered that the crystal growth is due to changing from two-dimensional to step-flow crystal growth by the misorientation angle and the direction with GaAs substrates. Quantum well structures with the thickness of several nm successfully enable being grown by the MOCVD method. This section describes o the development history of quantum well AlGaInP laser diodes and their characteristics. Furthermore, it introduces the recent progress of QD laser diodes and their future prospects.

#### 3.3.1. Development History of Quantum Well Laser

**Figure 6:**
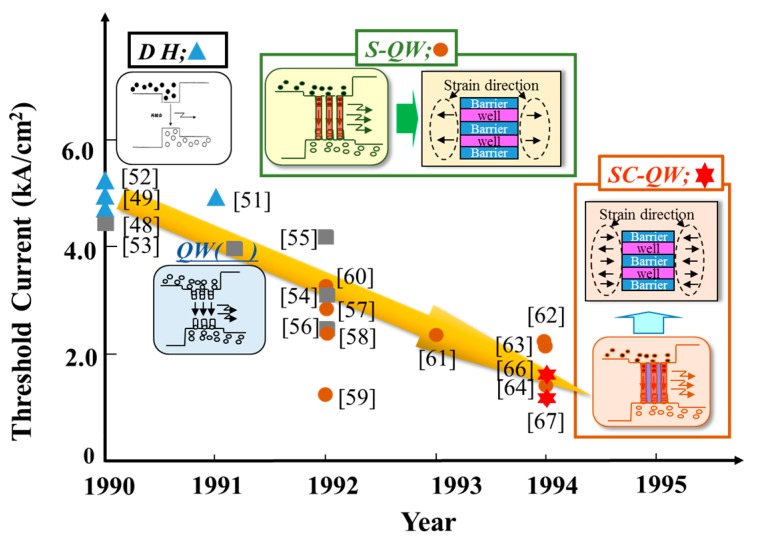
Development history of active layer for 630 nm band AlGaInP laser diodes with transverse mode stabilized structure. Optical guiding structure: optical loss guiding type [59]; Ridge structure [64]: *λ*/2-High Reflective (HR) facets coating laser.

Development of AlGaInP laser diodes and light emitting diodes (LEDs) were aggressively advanced from 1986 to 1996 [32]. Especially, threshold reduction of transverse mode stabilized 630 nm band laser diodes was achieved by introducing some kinds of quantum well structures, as shown in Figure 6. All laser diodes have loss-guided structures. This development has been mainly advanced using the double hetero structure (DH) with a bulky GaInP active layer since 1986.

**Figure 7:**
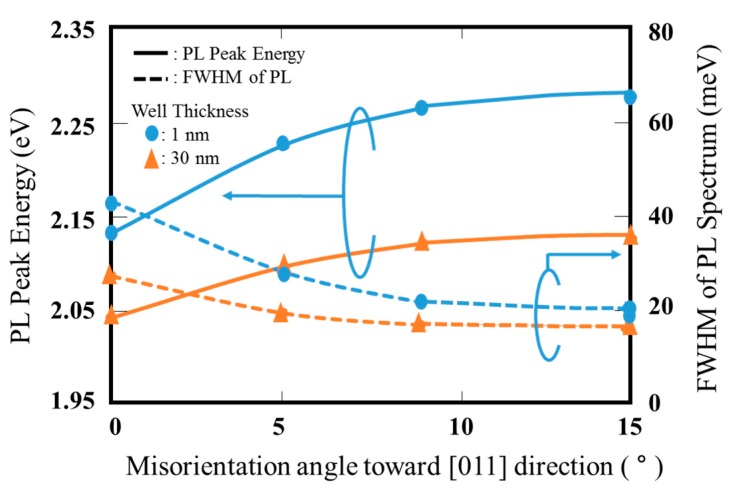
Relationship between PL peak energy and FWHM of PL spectrum as function of misorientation angle toward [011] direction.

The threshold current was about 100 mA at that time [48,49]. The reproducibility of 630 nm band laser diodes had been drastically improved by the crystal growth technologies using misorientation substrates discovered by our group in 1988 [50,51,52]. After that, the laser diodes, which are applied the multiple quantum well structure, were developed from 1990 to 1992 [53,54,55,56], and the threshold current is reduced by about 25% in comparison to that of lasers having a bulky active layer (DH). Then, to reduce the threshold current, strained quantum well lasers, such as circular symbols in Figure 7, were developed by many researchers [57,58,59,60,61,62,63,64]. In this development race, the world’s first high performance laser diodes with the strained compensated quantum well (SC-QW) structure, which adds compressively to the wells or tensile strain to the barriers, were developed by our group in 1992 [65]. The threshold current was one-half reduced in comparison to that of DH lasers. Then, our group successfully achieved threshold current reduction of about 75% in comparison to that of DH lasers by optimizing the strain balance in the well and barrier layers in 1994 [66]. Finally, the threshold current of 630 nm band laser diodes are as low as about 20.5 mA at 20 °C [67]. The laser diodes have been produced by many manufacturers since 1994. The next section describes the characteristics of the quantum well structure grown on misorientation substrates.

#### 3.3.2. Quantum Well Structure Grown on Misorientation Substrates

The interface between wells and barriers for the multiple quantum wells structure is, in general, very important for fabricating high performance devices. Figure 7 shows the relationship between the PL peak energy and the FMHM of PL emission spectrum for a GaInP single quantum well (SQW), as a function of the misorientation angle toward [011] direction. The barrier layers, which are also sandwiched, are (Al_0.5_Ga_0.5_)InP. The well thicknesses are shown for 1 and 3 nm. With an increasing of misorientation angle, the PL peak energy and the FWHM show an almost constant value, >9–10°. The thinner well is strongly affected by the misorientation angle of substrates. The results mean that the abrupt interface between the well and barrier is obtained using the substrates having misorientation angle of >9–10°.

**Figure 8:**
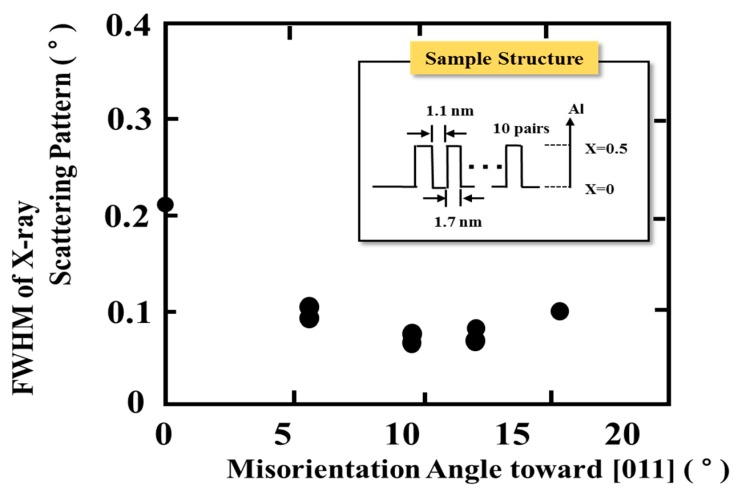
Relationship FWHM of the first peak in the small angle X-ray scattering pattern and misorientation angle toward [011] direction.

In this paper, the small angle X-ray scattering (SAXS) method is used to evaluate the periodicity and homogeneity in multiple-quantum well (MQW) structures. Figure 8 shows the relationship between the FWHM of the first peak of the SAXS pattern and the misorientation angle toward [011] direction. The thickness of a well and barriers are designed at 1.1 nm and 1.7 nm, respectively, as shown in Figure 8. The well and barrier layer are applied GaInP and (Al_0.5_Ga_0.5_)InP layer, respectively. The X-ray source is Cu K*α* radiation (*λ* = 0.1541 nm). The FWHM of SAXS pattern is dependent on the misorientation angle, showing a minimum value at the misorientation of 9°, as shown in Figure 8. It may be attributed to the phenomenon that the step-flow crystal growth is enhanced with an increasing of misorientation angle in the 0–9°, and crystal growth mechanism is changed from step-flow to two-dimensional growth at >9° [32]. The results are not consistent with those of Figure 7. This is based on the structure differences, such as the SQW and MQW. The compositional fluctuation at the interfaces between the wells and barriers are enhanced by the MQW having periodic structure. It is improved by optimizing of the growth conditions and misorientation angle.

**Figure 9:**
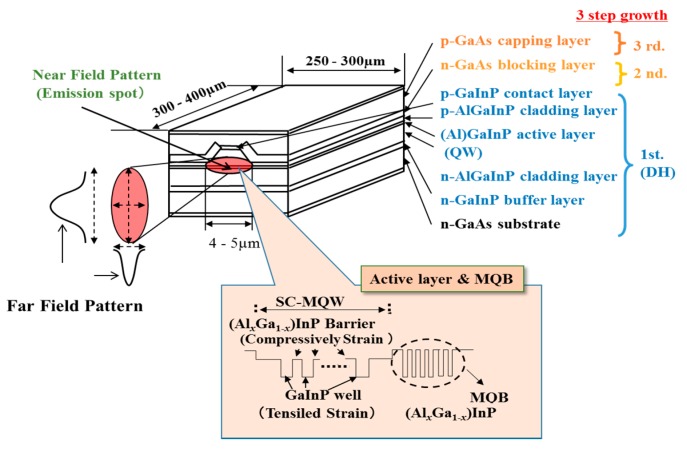
The 630-nm band AlGaInP laser diode, adapted from [65], with copyright permission from © 1992 IEEE.

Transverse mode stabilized laser diodes are shown in Figure 9 [65]. The structure is fabricated using the three-step MOCVD method. In the first stage, the DH structure was formed on GaAs (100) substrate with misorientation toward [110] direction. Next, the mesa stripe structure having a [01−1] direction is formed using the photolithography and the dry etching methods. At this time, the mesa stripes is formed using SiO_2_ mask. The current blocking layer is formed at the states that SiO_2_ mask remains on the top of the mesa ones. After that, the mask is removed by the wet etching processes, and p-GaAs contact layer is finally formed by the MOCVD method. After the thinning processes of GaAs substrates, the p- and n-type electrodes are deposited on the p-GaAs and n-GaAs substrates, respectively. The thinning process is needed to perform the cleave processes for forming the front and rear facets at high yield. Al_2_O_3_ passivation films are deposited on the front and rear cleaved facets. In the case of high power laser diodes, it is deposited the multilayered structure, which are alternatively stacked at thickness of *λ*/4 each film with low and high refractive index to prepare the rear facet with high reflectivity. Amorphous Si films are generally used as the high reflective index films. For front facets, it is coated Al_2_O_3_ film having low refractive index for reducing the reflectivity. The reflectivity of front facet is only controlled by Al_2_O_3_ film thickness. The films are deposited by the magnetron sputtering method. Then, each chip is cut out from the cleaved bar, and is mounted using the solder materials on the heatsinks such as Si and AlN, and the chips are settled the copper stem, which is mounted Si photodiode for monitoring the output power of laser, using low temperature solders. Finally, the stems are sealed by the metal cap with a glass window in the dry nitrogen ambient. The laser diode modules are widely used in applications for DVDs, displays, measurement tools, bar-code readers, and pointers. The temperature characteristics of 630 nm band laser diodes are not so good in comparison to those of 670–690 nm ones. Therefore, the laser diodes apply the multi-quantum barrier (MQB) structure, which was developed by Iga et al. [68]. The maximum operation temperature was improved up to 95 °C by the MQB. The threshold currents are reduced by introducing the real-index guide structure, and the characteristics are also improved. For 650 m band laser diodes with the structure, the threshold current of 8 mA is achieved at cavity length of 350 µm, and the maximum operation temperature is improved up to 120 °C. A lifetime of >3000 h is achieved at the operation conditions of 5 mW and 80 °C [69]. From these results, threshold current reduction of about 30% for 630 nm band laser diodes is estimated. The reliability of the laser diodes are affected by the temperature of active layer. Therefore, it is necessary to choose the heatsink materials for suppressing of the temperature rising in the active layer. Especially, AlGaInP laser diodes have essential issues that thermal conductivity of AlGaInP materials are about one-half lower than that of AlGaAs ones [70,71]. To improve the issues, Hamada et al. have chosen AlN ceramic heatsinks instead of Si heatsinks, which are used for AlGaAs laser diodes in 1991 [72].

Recently, aluminum nitride (AlN) heatsink is applied to high power AlGaInP laser diodes to achieve both low cost and high reliability performance [73]. The calculation method of the temperature rising in the active layer is described in Appendix A.

**Figure 10:**
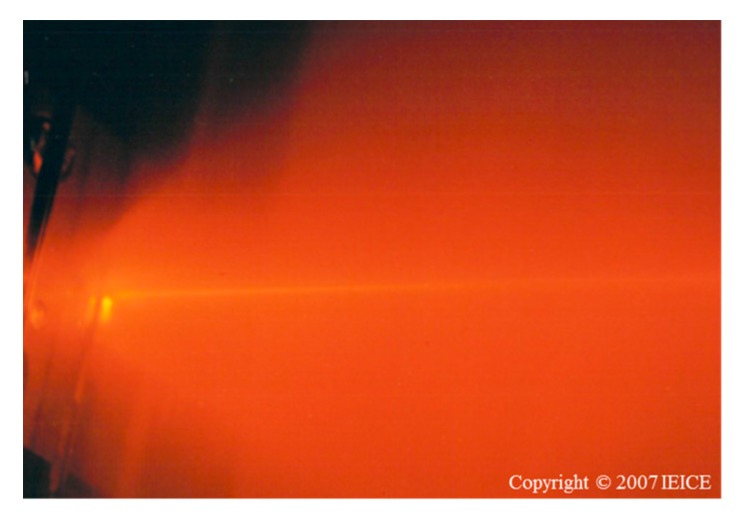
World’s shortest AlGaInP laser diodes (615 nm) oscillating at room temperature, adapted from [71], with copyright permission from © 2007 IEICE.

The world’s first 610 nm band laser diodes under CW operation have been successfully developed using compressively strained quantum well structures, MQB and misorientation substrates by Hamada’s team in 1992 [74].The oscillating state at room temperature is shown in Figure 10 [71]. After that, the laser diodes with tensile strain quantum wells have been also reported by Bour et al. and Tanaka et al. [74,75,76]. As a result, the limitation of CW operation at room temperature of AlGaInP laser diodes has been proven by the reports.

**Figure 11:**
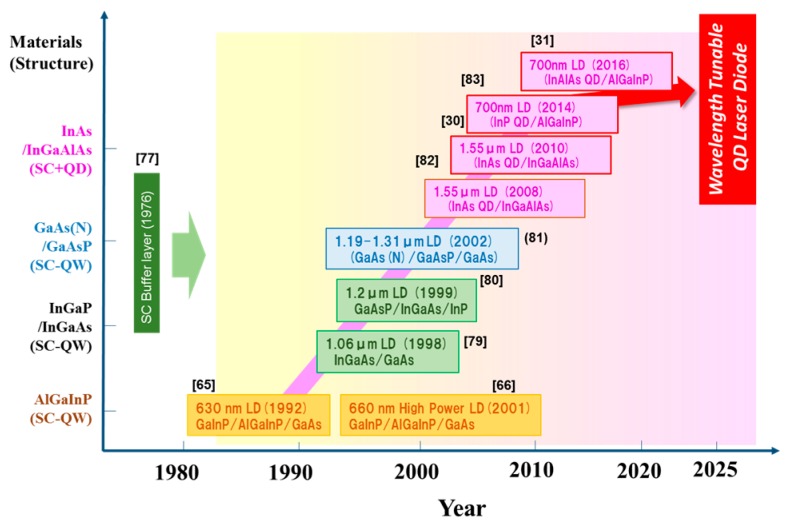
Development history of strain compensated quantum wells (QWs), quantum dots laser diodes, and their future prospects.

Figure 11 shows the development history of laser diodes based on the strain compensated quantum well (SC-QW) structures, quantum dots laser diodes and their future prospects. The strain compensated structure was developed as the buffer layer to obtain the high quality compound semiconductors by Matthews et al. in 1976 [77]. The SC-QW AlGaInP laser diodes have been developed by Hamada’s team in 1992 [65]. The threshold currents of the laser diodes are about one-half decreased in comparison to that of the strained quantum ones. Highly reliable laser diodes are also obtained by the structure [66,78]. The structure is applied to 1.0–1.2 µm band laser diodes for reducing the threshold current [79,80,81]. Then, laser diodes, which combine SC-QW and Quantum dots (QDs) structures to active layer, were developed in 2008 [82]. After that, wavelength tunable laser diodes capable of choosing the oscillating wavelength by changing the QD size were developed in 2010 [30], and the laser diodes, which were applied the different QD materials, have also developed [31,83]. The laser diodes have also the characteristics of high gain and temperature operation in comparison to the conventional ones. Therefore, the laser diodes will open the door of new applications such as in displays, communications, medical equipment, and sensing devices in the near future.

## 4. Summary

High quality GaInP epitaxial layers have been successfully grown on GaAs misorientation substrates using low-pressure MOCVD method. The ordered structure of GaInP epitaxial layers is controlled by the crystalline misorientation angle and the direction of the substrates. The ordered phenomena completely disappear using substrates having >9° misorientation toward [011]. On the other hand, highly ordered GaInP layers having an orientation toward [11−1] direction achieve using the substrates having 5° misorientation toward [01−1] direction. The bandgap energy of GaInP layers having completely disordered and highly ordered structures toward [1−11] direction are 1.14 eV and 1.791 eV, respectively. From the temperature dependence of PL spectrum, highly ordered GaInP layers are the same as the disordered ones, and are better than that of the ordered structure having two orientations. It is concluded that highly ordered epitaxial layers grown on the misorientation substrates toward [01−1] direction are essentially different from the ordered structures grown on GaAs (100) substrates, which were reported in the 1980s. The PL peak energy differences between the highly ordered and disordered GaInP epitaxial layers are about 130 meV at room temperature, which is the largest ever reported ones.

Furthermore, the strained compensated AlGaInP quantum well structure, which was proposed by Hamada’s team in 1992, have strongly contributed to developing and manufacturing laser diodes with low-threshold current. The threshold current of the transverse mode stabilized laser diodes are one-quarter lower than that of the double hetero structures with a bulky active layer. High performance 630 nm band laser diodes have been manufactured by applying the structure. The novel laser diodes, which introduce SC-QW and QD structures, will contribute to the development of the wavelength tunable ones, and open the doors of new applications.

## Figures and Tables

**Table 1:** Sample list of gallium indium phosphide (GaInP) epitaxial layers.

Sample NumberGaAs Substrate	Growth Temperature (°C)	Crystalline Structure		
		Ordered	Ordered + Disorded	Disordered
Ex144 (100) just	650	○ with two directions	-	-
Ex146 5° misorientation toward [011]	680	-	-	○ almost
Ex145-A 5° misorientation toward [011]	650	-	○ with week ordered	-
Ex145-B 5° misorientation toward [01−1]	650	○ with a single directions	-	-
Ex148 9° misorientation toward [011]	650	-	-	○ completely

**Table 2:** Activation energy for GaInP epitaxial layers.

Samples	Ca	Ea (meV)	Cb	Eb (meV)	Cb/Ca
Ex146	10^2.65^	24	10^4.3^	62	10^1.65^
Ex145-A	10^2.08^	24	10^4.0^	66	10^1.92^
Ex145-B	10^1.88^	16	10^4.0^	68	10^2.12^
Highly Ordered LC159 [36]	10^3.2^	16.4	10^1.46^	50	10^−1.74^

**Table 3:** Energy of variation mode of excited state (*ℏω*) and FWHM (*A*) of PL exported to 0 K for GaInP epitaxial layers.

Samples	*ℏω* (meV)	A (meV)
Ex146	7	7.9
Ex145-A	6.3	9
Ex145-B	4	12.4

**Table 4:** Characterization of ordered GaInP epitaxial layers.

Substrate	h (meV)	PL Peak Energy (eV) (at 300 K)	PL Peak Energy (eV) (at 10 K)	FWHM of PL stectrum (meV) (at 10 K)	Ref.
Ex145-B	8	1.79	1.85	12	-
(100) just	32	1.86 (at 290 K)	1.91	24 (at 6 K)	[43]
(100) just	12	1.83	1.9	-	[42]

**Figure A1:**
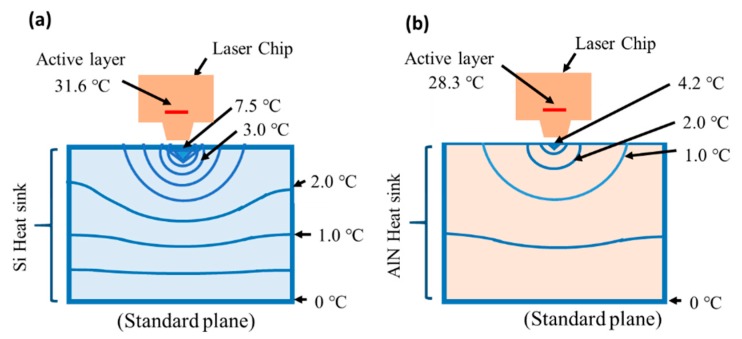
Simulated temperature of active layer: (**a**) Si heat sink; and (**b**) AlN heat sink.

## Appendix A

### Estimation Method of Temperature Rising of Active Layer

The thermal conductivity of AlN ceramic heatsink is 1.8 times higher than that of Si heat sink, as shown Table 4 [84]. To estimate the temperature of active layer, it is used the thermal conductive equation based on finite-element models as follows [32,85]:

(A1) $$\sigma (\partial^{2}/\partial X^{2}\;+\;\partial^{2}/\partial Y^{2}\;+\;\partial^{2}/\partial Z^{2})\;T\;+\;Q\;=\;0\;+$$

where *σ* and *Q* are thermal conductivity and calorific values, respectively. *T* is temperature rising. *X*, *Y*, *Z* show positional coordinates for lasers. The LDs chip is mounted on AlN ceramic heat-sink at junction down configuration. The temperature rising (Δ*T* °C) of active layer is three-dimensionally calculated using parameters in Table A1 and the following conditions:

1. Heat is only generated by injected electric power.
2. Thermal only flows from heatsink (thermal does not flow out from the laser chip to the atmosphere).
3. Thermal flow and temperature are continuous at interface of each layer.
4. Chip shape is mesa stripe structure.

**Table A1:** Parameters for calculation.

Prameters	Symbol	Value	Unit
Laser chip size	-	500 × 300 × 100	μm
AlN heat sink size	-	1100 × 1100 × 2400	μm
Operation current	*I_op_*	100	mA
Operation voltage	*V_op_*	2.5	V
Light output power	*P_out_*	30	mW
Thermal conductivity of GaInP	*σ_GaInP_*	0.053	W/cm·K
Thermal conductivity of AlGaInP	*σ_AlGaInP_*	0.06	W/cm·K
Thermal conductivity of GaAs	*σ_GaAs_*	0.44	W/cm·K
Thermal conductivity of Si	*σ_Si_*	1.45	W/cm·K
Thermal conductivity of AlN	*σ_AlN_*	2.6	W/cm·K

Temperature at each area in the laser chip is calculated by setting as 0 °C the temperature of heatsink back-side, as shown in Figure A1. The thermal conductivities of AlGaInP, GaInP and GaAs refer to data reported by Martin et al. [70].

The simulated results for Si and AlN ceramic heatsinks are shown in Figure 10. The temperature gradient in the AlN ceramic heatsink is lower than that of Si one. As a result, the temperature rising of active layer is effectively suppressed using AlN ceramic heatsink having the high thermal conductivity, and is reduced by 3.3 °C.

High performance transverse mode stabilized 630 nm band laser diodes have been successfully developed by introducing SC-MQW structure and AlN heatsinks having high thermal conductivity. The threshold current and maximum operation temperature of laser diodes with the cavity length of 350 µm and without facet coating are 20.5 mA (threshold current density; 1.1 kA/cm^2^) and 95 °C, respectively [67]. The laser diodes show the highest performance ever reported, to our knowledge.
